# Pretubulysin: From Hypothetical Biosynthetic Intermediate to Potential Lead in Tumor Therapy

**DOI:** 10.1371/journal.pone.0037416

**Published:** 2012-05-17

**Authors:** Jennifer Herrmann, Yasser A. Elnakady, Romina M. Wiedmann, Angelika Ullrich, Manfred Rohde, Uli Kazmaier, Angelika M. Vollmar, Rolf Müller

**Affiliations:** 1 Helmholtz Institute for Pharmaceutical Research Saarland, Helmholtz Centre for Infection Research and Department of Pharmaceutical Biotechnology, Saarland University, Saarbrücken, Germany; 2 Department of Pharmacy, Pharmaceutical Biology, Ludwig-Maximillians-University, Munich, Germany; 3 Institute for Organic Chemistry, Saarland University, Saarbrücken, Germany; 4 Department of Medical Microbiology, Helmholtz Centre for Infection Research, Braunschweig, Germany; Wayne State University School of Medicine, United States of America

## Abstract

Pretubulysin is a natural product that is found in strains of myxobacteria in only minute amounts. It represents the first enzyme-free intermediate in the biosynthesis of tubulysins and undergoes post-assembly acylation and oxidation reactions. Pretubulysin inhibits the growth of cultured mammalian cells, as do tubulysins, which are already in advanced preclinical development as anticancer and antiangiogenic agents. The mechanism of action of this highly potent compound class involves the depolymerization of microtubules, thereby inducing mitotic arrest. Supply issues with naturally occurring derivatives can now be circumvented by the total synthesis of pretubulysin, which, in contrast to tubulysin, is synthetically accessible in gram-scale quantities. We show that the simplified precursor is nearly equally potent to the parent compound. Pretubulysin induces apoptosis and inhibits cancer cell migration and tubulin assembly in vitro. Consequently, pretubulysin appears to be an ideal candidate for future development in preclinical trials and is a very promising early lead structure in cancer therapy.

## Introduction

Natural products play a very significant role in drug discovery and the development of novel pharmaceuticals, particularly anticancer compounds and antiinfective agents. The apparent drawbacks of these compounds, such as production, continuous supply, and the complex synthetic routes involved in natural product chemistry, are outweighed by the advantages of chemical diversity and numerous biological activities (‘privileged structures’).[1], [2]

Traditionally, the main sources of natural products have been soil bacteria, fungi, and higher plants, but in recent decades, cyanobacteria and marine organisms have also been of particular interest.[3] Among microbial sources, actinomycetes remain the best-studied organisms and represent a very rich source of bioactive secondary metabolites. Nevertheless, the success rates for the discovery of novel chemical entities from traditional sources with potent biological activities has decreased with time.[4], [5] Myxobacteria are increasingly recognized as proficient producers of bioactive secondary metabolites. This fascinating and widely expanding order of δ-proteobacteria has a unique and complex developmental life cycle,[6], [7] and in the last three decades, myxobacteria have become an outstanding source of natural products with unique structures, a broad spectrum of activities, and often completely novel mechanisms of action.[8], [9]

As reviewed by Newman and Cragg in 2012,[10] over 60% of anticancer drugs in the last 30 years have originated from natural products. Among the decisive cellular targets, microtubules have played a major role, especially since the Vinca alkaloids vinblastine and vincristine entered the market in the early 1960s. Moreover, chemotherapeutics belonging to this compound class continue to be highly valuable as part of the pharmacopoeia for cancer treatment.[11], [12] In mammalian cells, microtubules are crucial for trafficking, signaling, migration, and proliferation. These dynamic structures are composed of α,β-tubulin heterodimers that are constantly assembled and disassembled as the microtubules oscillate between growing and shortening phases (dynamic instability). Most importantly, the suppression of these dynamics by small molecules ultimately results in mitotic arrest and, in turn, the inhibition of cell proliferation and the induction of apoptosis. The search for novel microtubule-interacting compounds is primarily motivated by the need to overcome the typical resistance acquired to these drugs and the neurotoxicity of these compounds.[13]

To date, six distinct compound classes that are produced by myxobacteria have been found to directly interfere with the eukaryotic cytoskeleton by either stabilizing or destabilizing microtubules (epothilone [14], disorazol [15], and tubulysin [16]) or actin filaments (chivosazol [17], chondramide [18], and rhizopodin [19]). Of these, microtubule-targeting drugs have been the subject of concerted efforts to elucidate their biosynthesis, total synthesis, and semi-synthesis to improve their pharmacological properties and yields.[20], [21] Epothilone B, a microtubule stabilizer that acts in a manner similar to that of taxanes, is one of the highlights of myxobacterial natural product research; its semisynthetic analog, ixabepilone, was approved in 2007 for the clinical treatment of advanced breast cancer in the US.[22]

Tubulysins were first discovered by the bioactivity-guided screening of the myxobacterial strains *Archangium gephyra* Ar 315 and *Angiococcus disciformis* An d48. These compounds were described as very effective microtubule-destabilizing agents with structural similarity to dolastatin-10 and with GI_50_ values against mammalian cells in the picomolar to low nanomolar range.[16] The identification and molecular analysis of the biosynthetic gene cluster from *A. disciformis* An d48 revealed that the secondary metabolites are assembled by a PKS/NRPS (polyketide synthase/nonribosomal peptide synthetase) hybrid system that produces linear tetrapeptides consisting of N-methyl pipecolic acid (Mep) and isoleucine (Ile) followed by the unusual amino acid tubuvaline (Tuv) and an α-methylated aromatic γ-amino acid either derived from tyrosine (Tut, tubutyrosine) or phenylalanine (Tup, tubuphenylalanine). In this study of the biosynthetic machinery, a precursor of tubulysin was postulated for the first time; this precursor is released from the last module in the biosynthetic assembly line. The enzyme-free precursor, referred to as ‘pretubulysin’, would then undergo oxidation and acylation reactions to form the natural product.[23] In subsequent studies, pretubulysin was isolated in minute amounts from *A. disciformis* and shortly after, the total synthesis of the natural product was initiated. Thus, the biosynthetic hypothesis was finally proven by comparing synthetic and naturally occurring pretubulysin via mass spectrometry and NMR.[24]

Natural tubulysins can be obtained by fermentation in only limited amounts (several mg per L), and their isolation requires multiple chromatographic steps; both the fermentation and chromatography steps are expensive. As reported by other groups, the introduction of the rather labile N,O-acetal group at the N-terminus of Tuv is the most challenging synthesis step and limits production on a reasonable scale.[25], [26] Because of the lack of this structural moiety in pretubulysin, we are now able to synthetically produce pretubulysin D in multigram quantities and thus, circumvent the supply issues typical of most natural products in preclinical development. Although pretubulysin lacks the N,O-acetal and acetoxy functionalities of Tuv, these moieties do not appear to be crucial for biological activity, as determined by growth inhibition of cancer cell lines.[24], [27]

To date, no detailed study on pretubulysin's effects on cancer cell lines and purified tubulin has been published. We herein report the results of comparative biological studies on tubulysins, pretubulysin and one of pretubulysin's small precursors derived by chemical synthesis. We characterized the potential anticancer properties of these molecules in cell-based and in vitro systems, and our results strongly suggest that pretubulysin, as a more stable derivative of tubulysin, has characteristics that are very similar to those of the original natural products that have been associated with antimitotic effects. Our findings will aid the development of pretubulysin, or an even more simplified analog, as a novel antimitotic agent for tumor therapy.

## Results and Discussion

### Antiproliferative effects on tumor cell lines

**Figure 1:**
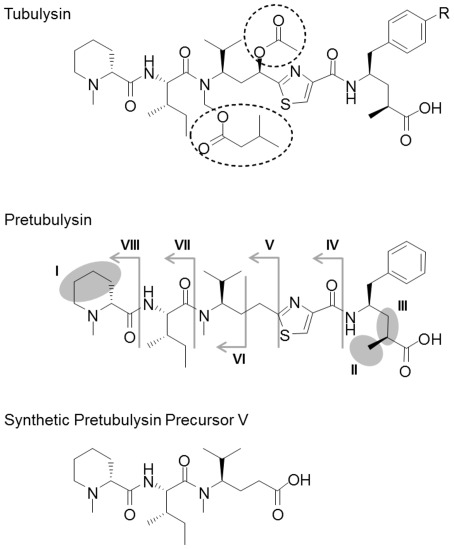
Structural comparison of tubulysin A (R = OH), tubulysin D (R = H), pretubulysin and a synthetic precursor. The modifications of all synthetic precursors of pretubulysin (I–VIII) are highlighted [I: Mep replaced by *N*-methylsarcosine; II: desmethyl Tup variant; III: missing propionate of Tup; IV: MepIleTuv; V: MepIleMe'Val' (γ-amino acid); VI: MepIleMeVal; VII: MepIle; VIII: Mep].

Our initial evaluation of the growth inhibition of cultured cells was based on tetrazolium salt reduction (MTT assay). Pretubulysin was active in a low to sub-nanomolar range against various tumor cell lines.[24], [27] Thus, based on the GI_50_ values, pretubulysin is, on average, only a factor of 10 or 100 less active than naturally occurring tubulysin A or D, respectively. We became especially interested in other even more simplified synthetic precursors (Figure 1 and S1) and their ability to inhibit the proliferation of cancer cell lines.

As reported earlier, the high potency of tubulysins is often unaffected by variations in the C-terminus, whereas N-terminal methylpipecolic acid (Mep) appears to be crucial for high potency.[28], [29] Here, tubulysins D and A exhibited GI_50_ values in the picomolar range against mouse L-929 cells, whereas pretubulysin was approximately 20-fold less active (5.8 nM). The removal of the C2-methyl group of Tup led to a further 10-fold reduction in activity (61.0 nM). Activity was nearly abolished in all other analogs. When N-Mep was replaced with N-methylsarcosine in the desmethyl derivative, a significant loss in activity was observed (8.5 µM). Variants in which the propionate group of Tup was missing (9.6 µM) or in which the C-terminal amino acid was missing (14.2 µM) were essentially inactive. Single Mep, MepIle, and MepIleMeVal building blocks did not inhibit L-929 cells at concentrations up to 25 µM. Interestingly, the MepIleMe'Val' derivative in which Val is replaced by the C2-prolonged homolog (γ-amino acid) (compound V, Figure 1) exhibited increased activity relative to MepIleTuv, with a GI_50_ value of 6.5 µM against L-929 mouse cells (Figure S1).

**Figure 2:**
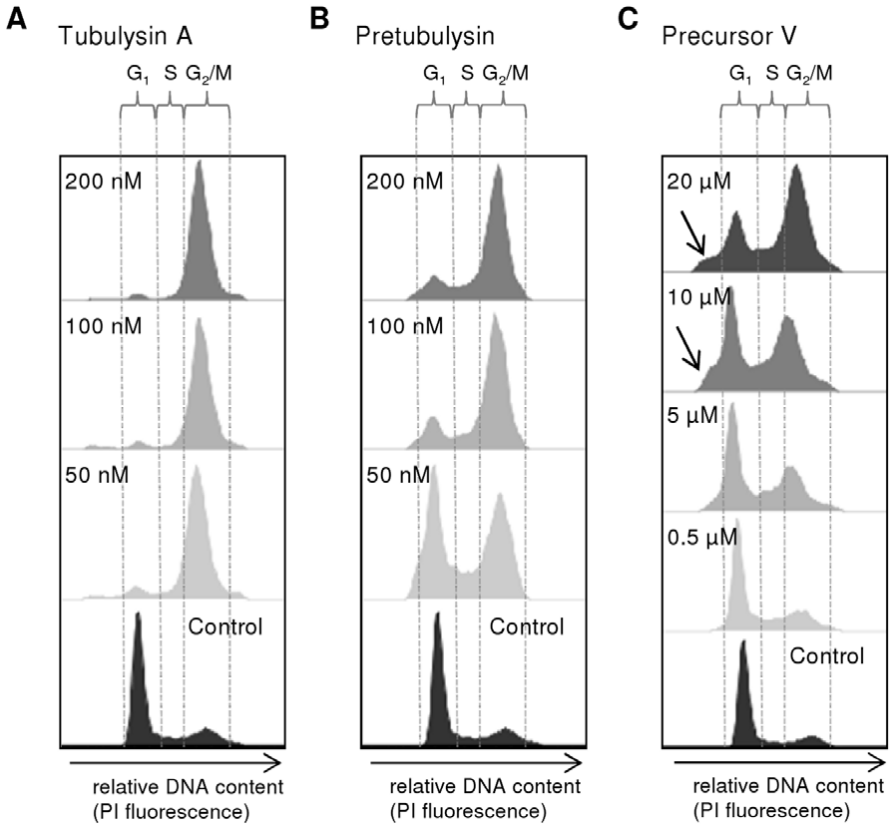
Comparison of induced G2/M cell cycle arrest in human HepG2 hepatocellular carcinoma cells. The cells were treated for 24 h with varying concentrations of **A**) tubulysin A, **B**) pretubulysin, or **C**) pretubulysin precursor V as indicated by cell cycle histograms. Fixed cells were analyzed after propidium iodide (PI) staining by flow cytometric measurements (FCM), and histograms of gated populations (excluding debris) were obtained by the Dean-Jett-Fox algorithm (quantitative data can be found as supporting material; Table S1). Histograms are representative results of a set of independent experiments. Arrows indicate the sub-G1 cell populations.

We next compared the potencies of tubulysin A and pretubulysin with respect to inducing the accumulation of cells in the G_2_/M phase of the cell cycle, which is a hallmark for tubulin-disrupting/antimitotic agents (Figure 2 and Table S1). As expected, pretubulysin is able to halt cells in the G_2_/M phase after 24 h of treatment. Similar to the results from the initial growth inhibition experiments with HepG2 cells, the antimitotic activity is comparable to that of the reference compound tubulysin A. Approximately 22% of the control cells are in G_2_/M phase. This population increases to approximately 73% or 82% after treatment with 50 nM or 200 nM tubulysin A, respectively; simultaneously, the G_1_ population decreases from 53% to less than 10% of the total number of measured cells (Figure 2A). Following a 24 h incubation with 50 nM pretubulysin, the cells are equally distributed in G_1_ (42%) and G_2_/M (44%) phases, and a more pronounced mitotic arrest with approximately 70% cells in G_2_/M phase is first observed at a concentration of 100 nM or greater (Figure 2B).

As determined by NMR structural analysis of tubulysin A bound to tubulin, the aromatic ring of Tut and the thiazole ring of Tuv form a basal platform in the tubulin-bound state that appears to be essential for full activity.[29] Although precursor V (Figure 1) lacks both aromatic rings, we could still observe the induction of G_2_/M cell cycle arrest in human hepatocellular carcinoma cells. At a final concentration of 5 µM, nearly 40% of all viable cells accumulated in G_2_/M phase. However, at higher drug concentrations, the percentage of viable cells in G_2_/M further increased to up to 60% in parallel with an emerging sub-G_1_ population, which most likely reflects late apoptotic cells in which the DNA has already been degraded (Figure 2C). This small pretubulysin precursor molecule is also likely able to destabilize microtubules to a certain extent and, in turn, leads to mitotic arrest in HepG2 cells. Nevertheless, the effect seems to be more nonspecific than that of pretubulysin itself, a difference that is reflected by a higher toxicity that is likely due to the greater than 100-fold-higher concentrations required to observe any effect on the cell cycle.

**Figure 3:**
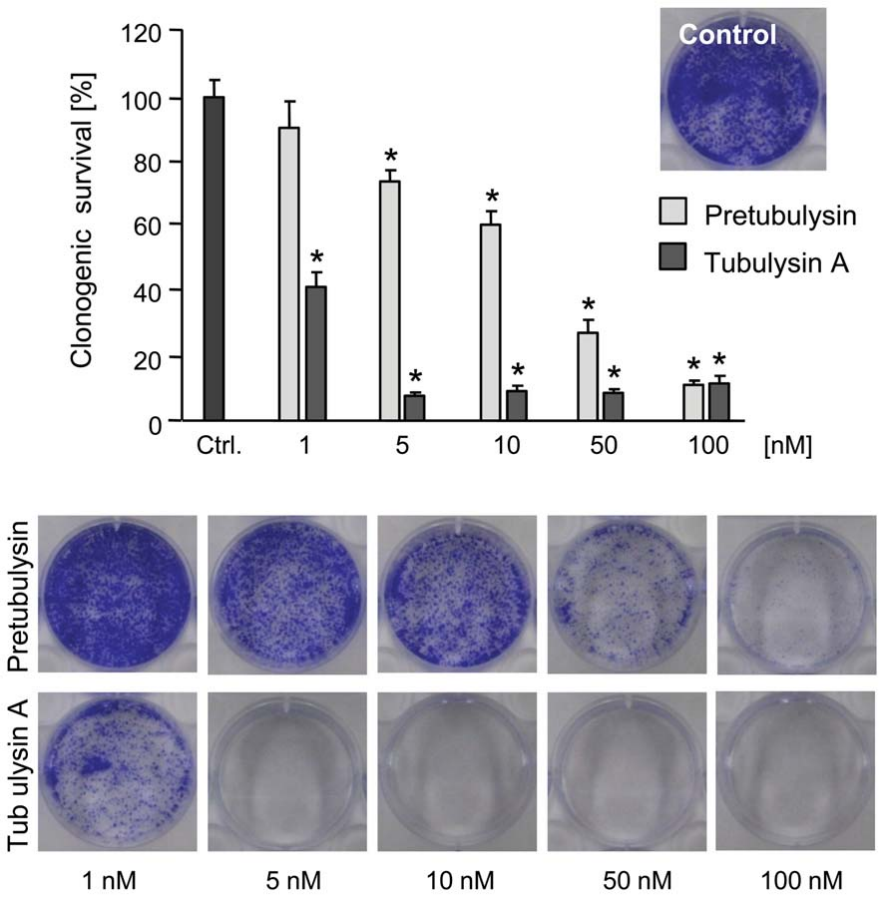
Tubulysin A and pretubulysin inhibit clonogenic survival of L3.6pl cells. L3.6pl cells were stimulated for 4 h and then seeded at low confluence. After 6 d, the cells were stained with crystal violet, and the absorbance was measured at 550 nm (n = 3; *p≤0.05 Anova/Dunnett).

Complementary to these results, we demonstrated that the long-term survival of human L3.6pl pancreatic cancer cells decreased after a 4 h exposure to tubulysins. Over a 6 d treatment period, the relative number of colonies formed by the cells was reduced to 40% of the control after pretreatment with 1 nM tubulysin A and to approximately 30% of the control after pretreatment with 50 nM pretubulysin (Figure 3).

### Apoptosis induction

**Figure 4:**
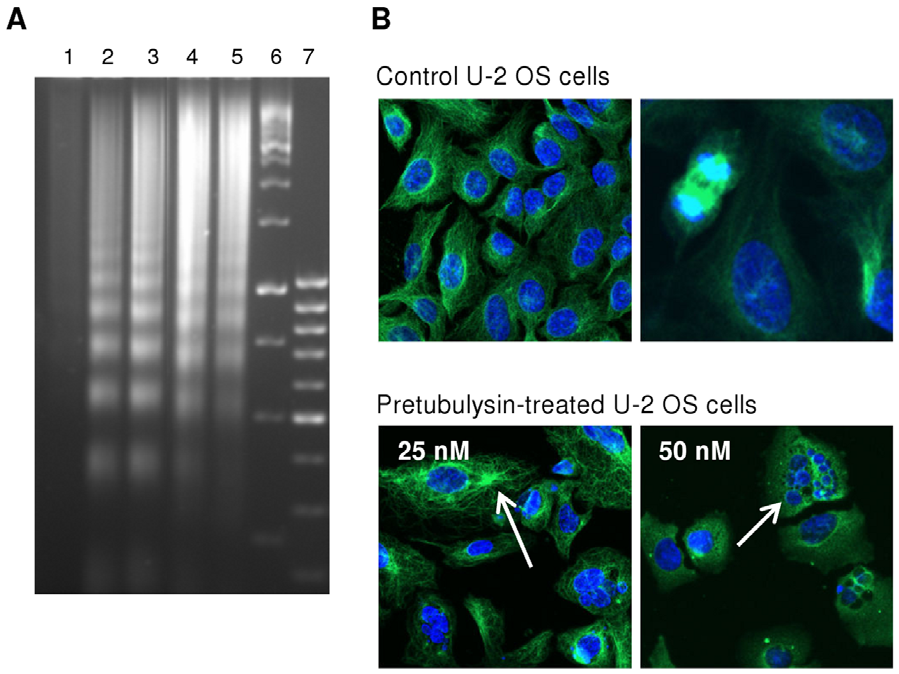
Typical cellular events upon pretubulysin treatment. **A**) Isolated DNA of human promyelocytic HL-60 cells treated with pretubulysin (1: control, 2: 30 nM, 24 h; 3: 75 nM, 24 h; 4: 30 nM, 48 h; 5: 75 nM, 48 h; 6: standard 1000 bp ladder; 7: standard 100 bp ladder). **B**) Human U-2 OS osteosarcoma cells that were left untreated (upper panel) or were treated for 24 h with pretubulysin (lower panel). Microtubules are visualized by immunofluorescence staining of α-tubulin (pseudocolor green), and nuclei were stained with Hoechst 33342 (pseudocolor blue). The arrows indicate the abnormal accumulation of microtubules in cells after treatment with 25 nM pretubulysin; fragmented nuclei, and completely depolymerized microtubules were observed at a drug concentration of 50 nM.

The aforementioned mechanisms of mitotic arrest are often found to trigger molecular signaling of the mitochondrial apoptosis pathway.[30] DNA fragmentation, effector caspase activation, and other regulatory events are useful parameters for gaining insight into the apoptotic mechanisms induced by microtubule-targeting compounds. Tubulysin A leads to DNA laddering in KB-3.1 cells,[31] and in this study, we clearly confirmed the same effect for pretubulysin at a comparable concentration range after a 24 h treatment (Figure 4A).

Antimitotic effects such as the formation of a multipolar spindle apparatus are believed to occur prior to the induction of apoptosis, which is accompanied by complete nuclear fragmentation and DNA laddering.[32], [33] All of these typical hallmarks occurred after treatment with pretubulysin. Treatment of human U-2 OS osteosarcoma cells with 25 nM pretubulysin resulted in partly fragmented nuclei and abnormal accumulation of microtubules around the periphery of the nuclei. At higher concentrations, microtubules were completely depolymerized, and most of the cells showed fragmentation of the nuclei (Figure 4B). At these pretubulysin concentrations, normal mitotic cells, which were present among the control cells, were no longer observed. Instead, cells with abnormal multipolar spindle pole structures were present (data not shown).

The proto-oncoprotein Bcl-2 (B cell lymphoma 2) is a key player in mitochondrial apoptosis because its phosphorylation status is linked to cell growth, the regulation of apoptosis, and the cellular redox status.[34] Inactivation, i.e., phosphorylation, of antiapoptotic Bcl-2 is accomplished by cellular mechanisms involving a variety of different kinases, such as JNK (c-Jun N-terminal kinase) and ERK1/2 (extracellular signal-regulated kinases).[35] The regulatory function of Bcl-2 can also be modified through the activation of pro-death molecules, such as epothilone B, which induces Bax (Bcl-2 associated X-protein) translocation to the mitochondria accompanied by cytochrome-c release into the cytosol, triggering the mitochondrial apoptosis pathway through the interaction of Bax with Bcl-2 and Bcl-xL.[36], [37]

**Figure 5:**
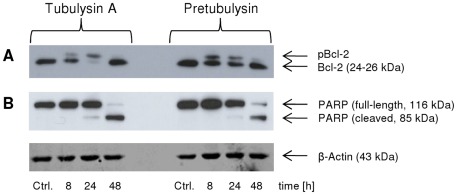
Immunoblot analysis of tubulysin A- or pretubulysin-treated L3.6pl pancreatic cancer cells. The cells were treated for the indicated time points with 10 nM tubulysin A or 10 nM pretubulysin. Lysates were immunoblotted for **A**) Bcl-2 and phosphorylated Bcl-2 (pBcl-2) or **B**) for PARP; β-actin served as a reference for the quantification of the protein levels. Ctrl.: lysates of untreated control cells.

We investigated the phosphorylation status of Bcl-2 in human L3.6pl pancreatic cancer cells and demonstrated that treatment with either tubulysin A or pretubulysin lead to transiently enhanced levels of phosphorylated Bcl-2 (pBcl-2) after 8 and 24 h treatments (Figure 5A). The highest pBcl-2 levels were detected after either 24 h of tubulysin treatment or 8 h of pretubulysin treatment. The initiation of Bcl-2 phosphorylation is a common characteristic of antimitotic drugs. Although the specific signaling events are only fairly understood, it is evident that MAPK activation during the course of treatment with tubulin-targeting agents is often linked to the phosphorylation status of Bcl-2.[38] Bcl-2 abrogates the release of cytochrome-c from mitochondria and subsequently the activation of caspase-9 and caspase-3.[39], [40]

**Figure 6:**
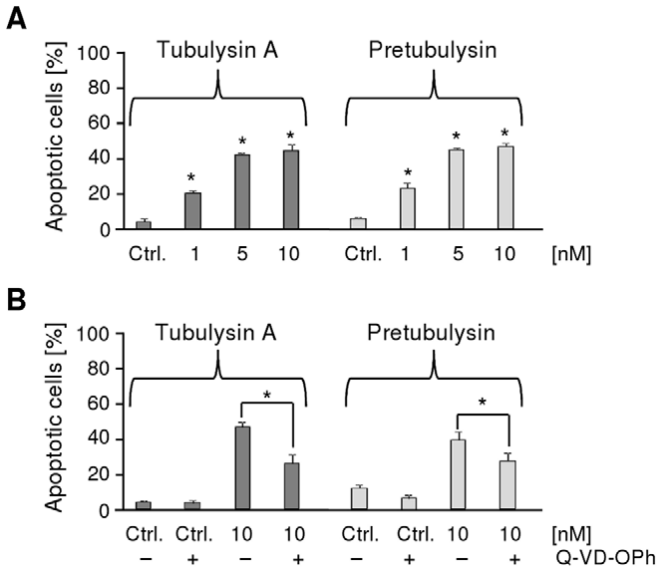
Induction of apoptosis in L3.6pl cells. The cells were treated for 48 h with **A**) varying concentrations of tubulysin A or pretubulysin (n = 2) or **B**) with 10 nM drug in conjunction with 10 µM caspase inhibitor Q-VD-OPh (n = 3). The percentages of apoptotic cells in the whole cell population were determined by PI staining (*p≤0.05 Anova/Dunnett).

Here, we demonstrated that treatment with pretubulysin and tubulysin A leads to the cleavage of poly(adenosine diphosphate-ribose) polymerase (PARP), which is a substrate of caspase-3 and functions in DNA damage detection and repair.[41], [42] PARP cleavage was detectable after a 24 h treatment with either drug, and this effect was even more pronounced after 48 h (Figure 5B). However, the apoptotic events initiated by tubulysin A and pretubulysin in cancer cells are only partly caspase-dependent. Treatment of L3.6pl cells with either compound resulted in approximately 50% apoptotic cells relative to the control as assessed by propidium iodide (PI) staining (Figure 6A). The broad-spectrum caspase inhibitor Q-VD-OPh reduced drug-induced apoptosis from approximately 46% to 26% and from 39% to 27% for tubulysin A and pretubulysin, respectively (Figure 6B).

### Comparative High-Content Screening (HCS)

In the course of HCS studies, we compared tubulysins with two other antimitotic compounds of myxobacterial origin, disorazol A and epothilone B, with a focus on microtubule-disrupting events. Disorazol A was originally isolated from the *Sorangium cellulosum* strain So ce12 [43] and was shown to be a highly potent cytostatic compound, with GI_50_ values against mammalian cells in the low picomolar range, by inhibiting microtubule formation.[44] Epothilone B was isolated from a *Sorangium cellulosum* strain (So ce90) due to its very high antifungal potential.[14] Epothilone B acts similar to taxanes by stabilizing microtubules.[45]

**Figure 7:**
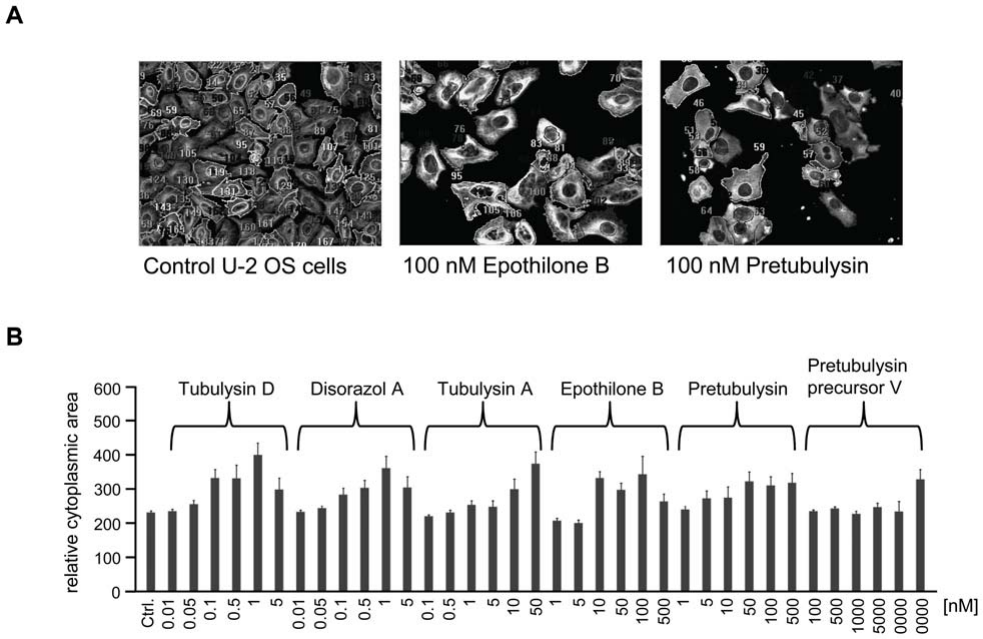
HC Analysis of the cytoplasmic area of U-2 OS cells treated with antimitotic compounds. **A**) Representative segmentation images of control U-2 OS cells and epothilone B- or pretubulysin-treated cells that were acquired with 200-fold magnification on a BD Pathway 855 automated microscope and subsequently processed and analyzed in AttoVision v1.6.2. **B**) Representative results of HC analyses of the average cytoplasmic area of U-2 OS osteosarcoma cells treated for 24 h with antimitotic drugs at varying concentrations. Bars represent the mean ± SEM of all cellular segments within a well.

**Figure 8:**
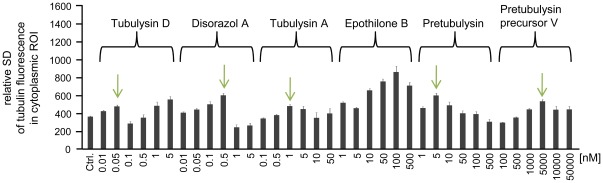
HC analysis of the standard deviation of tubulin fluorescence. Representative HCS results of several experiments on tubulin depolymerization. The SD is used as a proxy for the tubulin distribution within the cytoplasmic segment of U-2 OS osteosarcoma cells treated for 24 h with antimitotic drugs at varying concentrations. Arrows indicate peak SD values for depolymerizing agents due to the higher density of microtubules at intermediate drug concentrations. Bars represent the mean ± SEM of all cellular segments within a well.

For the HCS of human U-2 OS osteosarcoma cells, the final concentrations of the compounds were adjusted based on their growth inhibitory potential, and cells were treated for 24 h. α-Tubulin was probed by immunofluorescence, nuclei were stained with Hoechst 33342, and HCS CellMask™ Red stain was added to facilitate subsequent segmentation of the cytoplasmic region. The most obvious features were the enlargement of cells, the fragmentation of nuclei, and the rearrangement of the microtubule structure. Analyses were thus performed on a single-cell basis and averaged over the complete sample. In a supervised, but unbiased approach we found that standard deviation of tubulin fluorescence and shape parameters of the cytoplasmic segments are the most suitable to describe antimitotic phenomena. This was done by experimentally creating a test set and classification in six cellular states. All fluorescence parameters that can in principle be investigated using the automated microscope were extracted, and important features were defined and cross-validated by using statistical methods (J. Herrmann, R. Müller et al., unpublished results). Certain characteristic parameters for every cellular change were then calculated within either the nuclear or cytoplasmic segments. Additionally, the standard error of the mean (SEM) was calculated for average values derived from single cells within a well. Throughout all HCS experiments the SEM values increased with increasing concentrations of the compounds, which is due to larger numbers of apoptotic cells and therefore more heterogeneous cell populations mainly with regards to cell size.In accordance with the growth inhibition induced by all of the tested compounds, the cell bodies were significantly enlarged in a concentration-dependent manner (Figure 7). This cytopathic effect is common to many cytostatic compounds and is particularly characteristic of antimitotics. Thus, epothilone B, a microtubule stabilizer, yields the same macroscopic characteristics as the other microtubule-depolymerizing compounds in the test panel. Upon treatment with the reference compounds tubulysin D, disorazol A, and epothilone B, a slight drop can be observed at the highest concentration tested, which is due to rounded-up, late apoptotic cells. In addition, the standard deviation (SD) of microtubule fluorescence, which serves as a measure of the tubulin distribution within cells, peaks at the intermediate concentrations used in the HC assays for all microtubule-destabilizing compounds (Figure 8).

**Figure 9:**
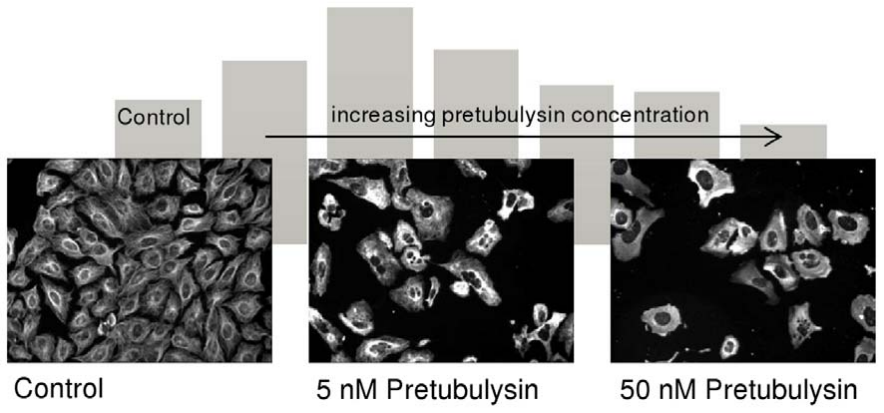
HC images of control U-2 OS cells and cells treated with pretubulysin. α-Tubulin was visualized by immunofluorescence, and images were acquired with 200-fold magnification on a BD Pathway 855 automated microscope. The background shows the details of the HC analysis of the SD of tubulin fluorescence (Figure 8).

In our hands, the SD of tubulin fluorescence has shown to be a robust measure to describe microtubule disruption in various assays. The more uniform tubulin appears in the cells, the lower the SD is, i.e., the lowest SD values are observed for cells with completely depolymerized microtubules. We observed a maximum value at intermediate concentrations, followed by a decline at higher concentrations; for tubulysin D, a second peak was observed at the highest concentrations due to cell debris. Upon image data dereplication (Figure 9), it was apparent that an increase in the tubulin fluorescence SD and the peak SD are linked to the initial depolymerizing effect, which is characterized by a greater number of mitotic cells with abnormal spindle pole structures and a higher density of interphase microtubules around the periphery of the cells. Increasing concentrations then led to the complete depolymerization of microtubules, as indicated by the lower SDs of the tubulin fluorescence. These characteristics were evident for all tubulin-destabilizers and, most interestingly, for the small pretubulysin precursor V. In summary, we assume that the dynamics of pretubulysin-induced microtubule depolymerization are highly comparable to those observed with the reference compounds tubulysin A/D and disorazol A. For epothilone B, we basically observed a continuous increase in the tubulin fluorescence SD due to the stabilizing effects of this compound. At the highest epothilone B concentration the fluorescence SD slightly falls off, which is most likely due to the very pronounced microtubule-stabilizing effect.

**Figure 10:**
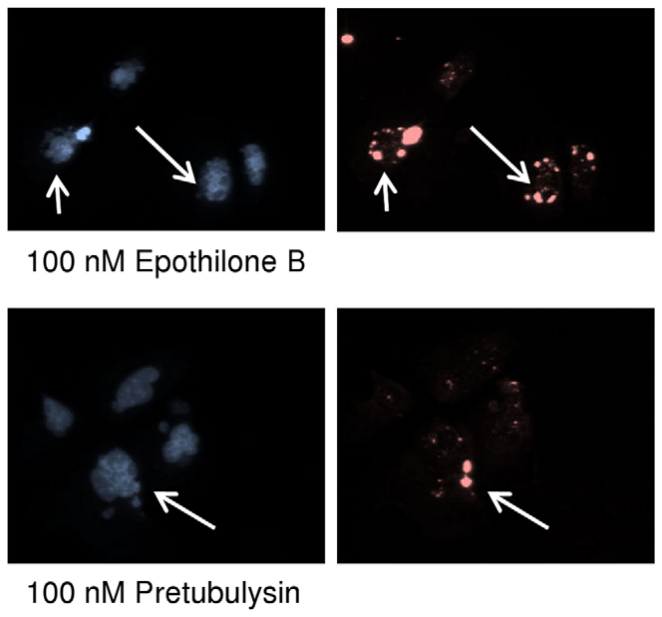
Representative images of U-2 OS cells treated with 100 nM epothilone B or pretubulysin for 48 h. Nuclei were stained with Hoechst 33342 (pseudocolor blue, left panel), and γH2A.X was visualized by immunofluorescence (pseudocolor red, right panel). Arrows indicate fragmented nuclei with bright fluorescent spots due to H2A.X phosphorylation events. Images were acquired on a BD Pathway 855 automated microscope and subsequently processed and analyzed in AttoVision v1.6.2 (the full HCS analysis is provided as supplementary material, Figure S2).

The analysis of nuclear fragmentation revealed a concentration-dependent increase in the nuclear fluorescence intensity accompanied by a higher variability in the intensities within single nuclei (data not shown). In a more detailed study on DNA double-strand breaks (DSBs), we found that all tested drugs led to large γH2A.X foci that were exclusively associated with already fragmented nuclei. Phospho-histone γH2A.X is widely used as a marker for DSBs because H2A.X is phosphorylated on serine residue 139 within seconds after the induction of DSBs in mammalian cells.[46], [47] Here, we treated the cells as in the microtubule HCS but for 48 h instead of 24 h. The cells were segmented on nuclear ROIs (regions of interest), and the immunofluorescence of γH2A.X was measured (Figure S2). Our results suggest that the tested drugs do not directly induce DSBs at sublethal concentrations but initiate downstream signaling, finally leading to enhanced H2A.X phosphorylation, which can only be found in at least partially fragmented nuclei (Figure 10).

### Inhibition of cancer cell migration

Tumor cell migration is directly linked to metastasis and is one of the most promising targets in cancer therapy; the inhibition of tumor cell migration may decrease the metastatic capacity of solid tumors. [48], [49]

**Figure 11:**
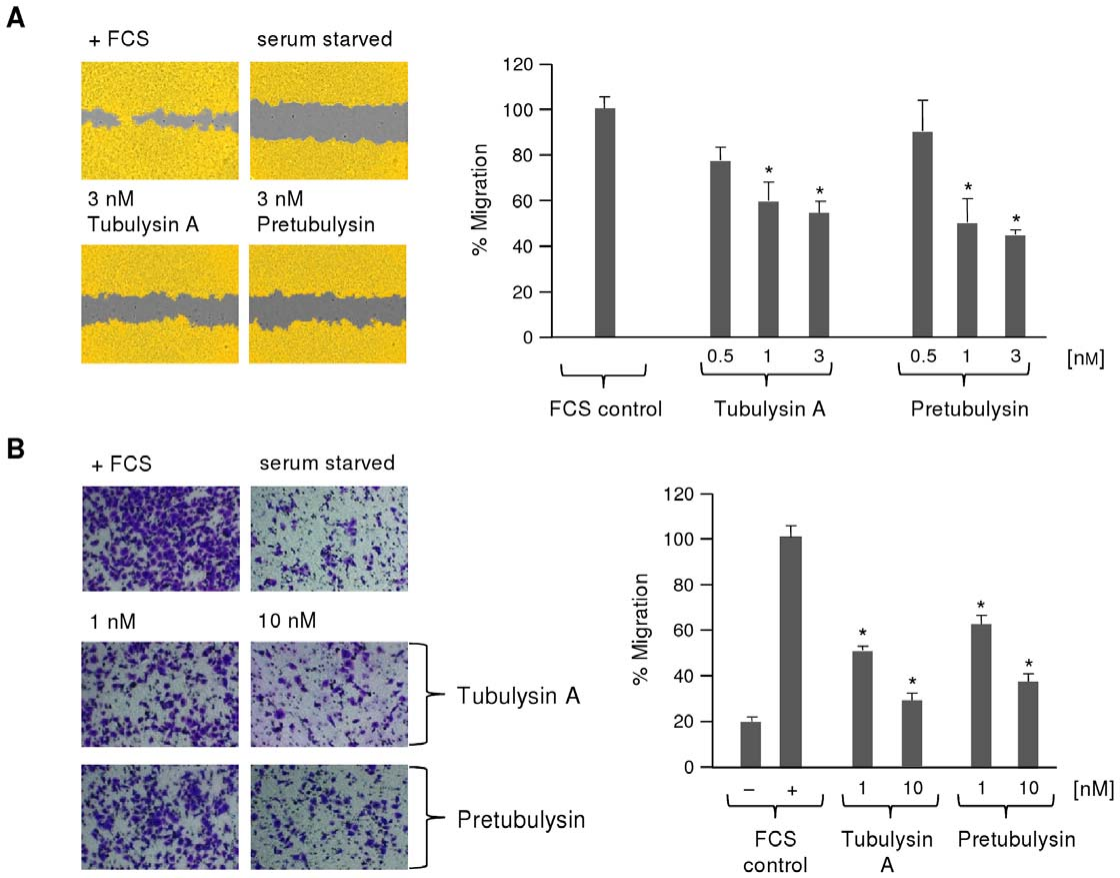
Inhibition of L3.6pl pancreatic cancer cell migration by pretubulysin and tubulysin A. **A**) ‘Wound healing’ assay. A confluent layer of L3.6pl was scratched and either left untreated or stimulated with varying concentrations of drug (n = 2). Left panel: Regions covered with cells are shown in yellow, and uncovered areas are displayed in gray. Right panel: The percentage of migration is expressed relative to the starvation control (0%) or the untreated control containing FCS (fetal calf serum; 100%). **B**) Boyden chamber assay with L3.6pl cells that were left untreated or stimulated with 1 or 10 nM drug (n = 2). Cells migrating toward a FCS gradient were stained with crystal violet (left panel), and the percentage of migration (right panel) was expressed relative to the levels of migration for the negative controls with (100%) or without FCS (*p≤0.05 Anova/Dunnett).

In our studies on tumor cell motility, we used the metastatic human L3.6pl pancreatic cancer cell line. The stimulation of L3.6pl cells with a 3 nM concentration of either compound led to a significant reduction in migration in scratch assays (Figure 11A) to approximately 50% of the cell-covered area relative to the untreated control population (FCS supplemented; 100%). In addition, treatment with 1 nM tubulysin A or pretubulysin reduced the relative number of migrated L3.6pl cells to approximately 40% of the control value (FCS supplemented; 100%) in a Boyden chamber assay. The number of migrating cells could be even further reduced to 30% of the control at drug concentrations of 10 nM (Figure 11B). It is hereby noticeable, that inhibition of migration is not due to apoptotic events after treatment with pretubulysin or tubulysin, as apoptosis induction in L3.6pl cells was seen as late as 48 h after treatment with 1 nM of either tubulysin A or pretubulysin. This treatment led to only 20% apoptosis induction in L3.6pl cells (Figure 6).

### In vitro studies on tubulin

Naturally occurring tubulysins A-I differ by up a factor of 60 in their inhibitory potency against L-929 mouse fibroblasts. Potency is thought to be primarily influenced by the lipophilicity of the compound. Tubulysin D is the most lipophilic, and thus, is also the most active of the derivatives. These findings are highlighted by the results of in vitro tubulin polymerization studies that showed only minor differences in the overall activities of the tested tubulysins.[50] We were also interested in comparing the in vitro activities in a cell-free system of tubulysin A, pretubulysin, and pretubulysin's small precursor V to elucidate whether the synthetic pretubulysins exhibit similar tubulin-binding potencies without the cell membrane acting as a natural barrier.

**Figure 12:**
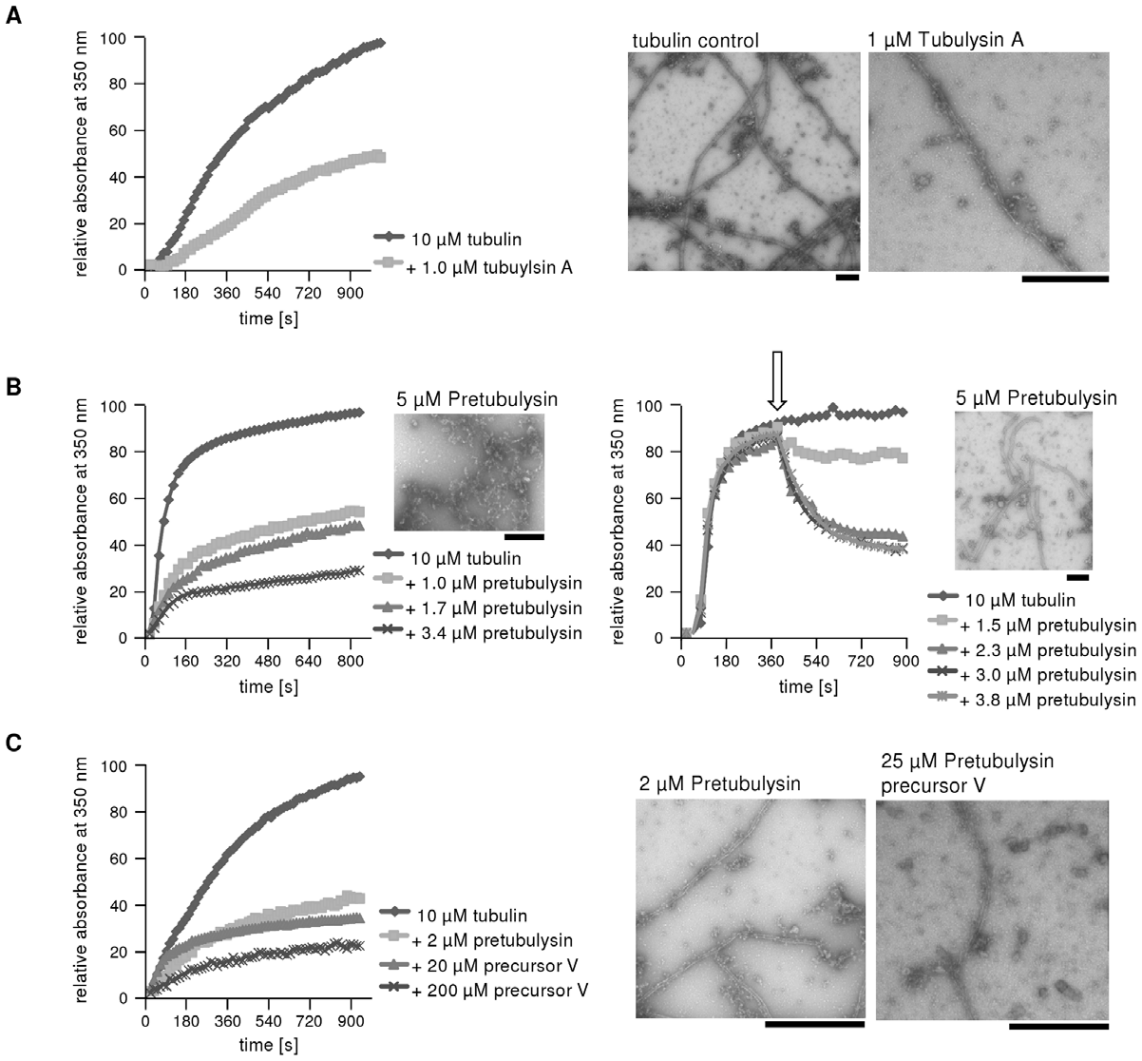
In vitro inhibition of tubulin polymerization by tubulysin A, pretubulysin and a small precursor of pretubulysin. **A**) As a reference, 1 µM tubulysin A was added to a preparation of 10 µM tubulin, and the absorbance was measured over time at 350 nm; the maximum value of the untreated control was set to 100%. Electron micrographs of the corresponding samples are shown on the right. **B**) Pretubulysin was either added pre-assembly (left panel) or post-assembly (right panel; the arrow indicates the time at which the drug was added) to a preparation of 10 µM tubulin, and the absorbance was measured at 350 nm; the maximum value of the untreated control was set to 100%. The electron micrographs in the upper corners display different degrees of disruption for both treatment types caused by pretubulysin. **C**) Tubulin protein was treated pre-assembly with either pretubulysin or its precursor, and the absorbance at 350 nm was measured and then expressed relative to the absorbance of the tubulin control (100%). Electron micrographs show disrupted microtubules upon treatment with pretubulysin or its precursor V. The scale bar on each micrograph is 500 nm.

As a reference, we recorded the absorbance of microtubule protein at 350 nm over time for 10 µM tubulin and demonstrated that the addition of 1 µM tubulysin A led to an approximately 50% reduction in built-up microtubules (Figure 12A). The corresponding electron micrographs reveal perfect tubular structures and long microtubules in the control. Upon the addition of tubulysin A, a smaller number of shorter and disrupted microtubules were observed. Similar to previous studies on tubulysin A [31], we demonstrated that pretubulysin can inhibit microtubule formation in vitro in a dose-dependent manner and can also depolymerize already assembled microtubules (Figure 12B). The addition of 1 µM pretubulysin (pre-assembly) led to an approximately 50% reduction in microtubule formation, whereas approximately 2 µM pretubulysin was needed to reduce the number of already built-up microtubules to the same extent. Electron micrographs of 12 µM tubulin incubated with 5 µM pretubulysin revealed that higher concentrations of the drug are needed to fully destroy microtubules rather than inhibit assembly. The pre-assembly addition of pretubulysin fully inhibited the polymerization process because only single protein blocks were found. In contrast, the post-assembly addition did not lead to complete depolymerization; thus, shorter and disrupted microtubule structures were observed.

Furthermore, we studied the effect of pretubulysin precursor V (Figure 1) and observed an approximately 10-fold reduction in activity as measured by the in vitro inhibition of tubulin polymerization (Figure 12C). Electron micrographs revealed that treatment with either 2 µM pretubulysin or 25 µM pretubulysin precursor V yields similar results with respect to microtubule disruption, as depicted by the shortened microtubules and the perturbed tubular structure relative to the control.

Taken together, our results suggest that pretubulysin is comparably potent to the parent tubulysin A with respect to microtubule disruption in vitro. Thus, we assume that pretubulysin has a similar binding affinity. The small precursor V was significantly less active, suggesting not only that cellular uptake or reduced metabolic stability causes the loss of growth inhibition activity but also that the binding affinity to purified tubulin is reduced.

### Conclusion

We demonstrated that synthetically accessible pretubulysin exhibits only a minor reduction in potential anticancer activity relative to the parent compound tubulysin, as seen in cell-based and in vitro studies. With a synthetic route in hand, pretubulysin or its derivatives appear to be better-suited for the development of novel antimitotic agents in tumor therapy, including tumor-targeting constructs, because the supply issues commonly encountered in the preclinical development of pharmaceuticals from natural products have been circumvented. Studies on further simplified pretubulysins are currently ongoing.[51] Although the activity of a direct synthetic precursor of pretubulysin was reduced, we demonstrated that even a very small molecule (precursor V), consisting only of two out of the four original amino acids, can bind to tubulin in vitro and cause microtubule depolymerization in cell-based assays. Thus, we believe that pretubulysin can be easily modified by means of further simplification and the attachment of various functional groups. Future synthetic approaches will thereby aid in obtaining greater insight into the mode of action of this compound family and will allow the ‘fine-tuning’ of the target binding, toxicity, and tumor specificity properties of pretubulysin.

## Materials and Methods

### Compounds and chemicals

The reference compounds epothilone B, disorazol A, and tubulysins A and D were kindly provided by the Microbial Drugs research group (MWIS) at the Helmholtz Centre for Infection Research (HZI, Braunschweig, Germany). All chemicals were of reagent grade quality and were obtained from commercial sources and used without further purification.

### Cell cultures

The L3.6pl cell line [52] was kindly provided by C. J. Bruns (Department of Surgery, Clinic Großhadern, LMU Munich, Germany). All other cell lines were obtained from the American Type Culture Collection (ATCC) and the German Collection of Microorganisms and Cell Cultures (Deutsche Sammlung für Mikroorganismen und Zellkulturen, DSMZ). All cell lines were cultured under the conditions recommended by the respective depositor. L3.6pl cells were cultured in RPMI medium supplemented with 10% FCS, 1 mM pyruvate, 1% non-essential amino acids) and cultureware was coated with collagen G (0.001% collagen/PBS). Media were purchased from Sigma or PAA, fetal bovine serum (FBS) and non-essential amino acids were from PAA, pyruvate was purchased from Sigma, and other reagents were from GIBCO (Invitrogen). Plasticware was obtained from Nunc, Sarstedt, BD Biosciences, and Corning.

### Cell cycle analysis

Cells were seeded in 6-well plates in 5 mL of complete medium and, after an o/n equilibration, were treated with the compounds dissolved in methanol at the appropriate concentrations and for the time periods indicated in the text. Approximately 10^6^ cells were harvested by centrifugation, washed twice with PBS (phosphate-buffered saline, pH 7.4) and fixed in cold (−20°C) 80% methanol. After an o/n fixation at −20°C, the cells were washed with PBS and further permeabilized with 0.1% saponin in PBS. For DNA staining, the cells were resuspended in staining solution containing 20 µg mL^−1^ propidium iodide (PI) and 100 µg mL^−1^ ribonuclease A (RNase A) in PBS. The cells were stained for 30 min at 37°C and analyzed on a GUAVA EasyCyte Plus flow cytometer (Millipore). In total, 10^4^ events, excluding cell debris, were measured, and the data were processed using FlowJo v7.6.5 software (Tree Star Inc.). For cell cycle analysis, the Dean-Jett-Fox algorithm was used.

### Clonogenic survival

L3.6pI cells at 70% confluence were treated for 4 h. After the treatment, the cells were trypsinized, and 10^4^ L3.6pl cells were seeded in a 6-well plate. The freshly seeded cells were allowed to grow for 6 d, followed by crystal violet staining for 10 min. Pictures of the wells were taken, and the crystal violet was redissolved with sodium citrate solution to measure absorption at 550 nm in a SpectraFluor Plus™ (Tecan, Männedorf, Austria). For statistical analysis, the percentage of viable cells for untreated cells was set to 100%.

### DNA ladder detection

DNA fragments were extracted from apoptotic cells in phosphate-citrate buffer (PCB, pH 7.8) as described by Gong et al.[53] In brief, cells were harvested by centrifugation and fixed o/n at −20°C in 70% ethanol. The extraction was performed by resuspension in PCB buffer, which consisted of 24 parts 0.2 M Na_2_HPO_4_ and 1 part 0.1 M citric acid. The supernatant was then treated with 0.25% Nonidet NP-40, RNase A, and proteinase K. The extracted DNA was loaded onto an agarose gel (1.7%) and separated by 4 h of electrophoresis at 4 V/cm using 0.5× TBE buffer (45 mM Tris-borate, 1 mM EDTA). The resulting bands were stained with ethidium bromide and detected under UV light.

### Immunoblotting

Proteins were separated by SDS-PAGE and transferred to nitrocellulose membranes via tank blotting. Nitrocellulose membranes were incubated with antibodies raised against Bcl-2 (Calbiochem), PARP (Calbiochem) or actin (Millipore). To detect the protein levels, the ECL™ detection system (Amersham Pharmacia Biotech, Little Chalfont, UK) or the Odyssey Infrared Imaging system version 2.1 (LI-COR Biosciences, Lincoln, NE, USA) was used.

### Apoptosis measurement

Apoptotic cell death was measured as described by Nicoletti et al.[54] Briefly, cells were permeabilized with a Triton-containing buffer and stained with 50 µg mL^−1^ propidium iodide. The cells were analyzed by flow cytometry.

### HCS studies

Human U-2 OS osteosarcoma cells were seeded at 5×10^3^ cells per well in 96-well imaging plates (BD Falcon). After an o/n equilibration, the cells were treated with antimitotic drugs, reference drugs, and a solvent control at the appropriate concentrations and treatment periods indicated for each experiment. The cells were fixed with cold (−20°C) acetone/MeOH (1∶1) for 10 min. After washing with PBS, the cells were permeabilized with 0.01% Triton-X 100 in PBS. The following combinations of primary and secondary antibodies were used: α-tubulin mAb (1∶2000, Sigma)/goat-anti-mouse-Alexa 488 (1∶1000, Molecular Probes) and phospho-Histone γH2A.X (Ser139) mAb (1∶400, Millipore)/goat-anti-mouse-Alexa 488 (1∶1000, Molecular Probes), each diluted in PBS/10% FBS. For labeling, cells were incubated with primary antibody for 45 min at 37°C, followed by incubation with the secondary antibody under the same conditions. The nuclei were stained with Hoechst 33342 (5 µg mL^−1^; Molecular Probes), and a whole cell stain (1 µg mL^−1^; HCS CellMask™ Red stain, Invitrogen) was used to facilitate later cell segmentation. The samples were imaged on an automated microscope (BD Pathway 855) suitable for high-content screening with appropriate filter sets for Alexa 488, Hoechst, and CellMask™ Red fluorescence. For the analysis of microtubules, a dual mask for the cytoplasm and nuclei was used in the CellMask™ Red and Hoechst channels, respectively, and the assigned parameters were calculated in either one of these ROIs as indicated. For the DSB analyses, a polygon nuclear mask was used, and γH2A.X fluorescence was calculated within this ROI. The average values per well are given.

### Scratch assay

L3.6pl cells were seeded 1 d before stimulation. The confluent cell layer was scratched, and tubulysin/pretubulysin that had been diluted in culture media was added. Scratched and treated cells were incubated at 37°C for 16 h. Cells were fixed with 3% PFA, and images were taken using a microscope (Axiovert, Zeiss). The analysis was performed with WimScratch software (Wimasis, Munich, Germany).

### Migration assay (Boyden chamber)

For each Boyden Chamber, 10^5^ L3.6pl cells were resuspended in media without FCS and added on top of the Boyden chamber membrane. Tubulysin/pretubulysin was also added to this suspension. On the bottom of the Boyden chamber membrane, culture media containing 10% FCS+100 ng mL^−1^ EGF was added. During cell migration, the chambers were incubated at 37°C in 5% CO_2_ for 16 h. Migrated cells were fixed and stained with crystal violet/methanol. Non-migrated cells on the top of the chamber were removed with a cotton swab. Images were acquired, and the migrated cells were counted and normalized relative to control cells, which represent 100% migration.

### Tubulin isolation

Microtubule proteins were isolated from porcine brain tissue as described elsewhere.[55] In brief, brains were homogenized in PEM buffer (0.1 M PIPES, pH 6.6; 1 mM EGTA; 1 mM MgSO_4_) in the presence of 0.1 mM GTP and 2.5 mM ATP, followed by three cycles of temperature-dependent depolymerization and polymerization. Purified tubulin was either stored at −80°C or directly used in turbidimetric assays.

### Tubulin polymerization assays

Tubulin polymerization was analyzed in a turbidimetric assay.[56] The samples (200 µL, 10 µM tubulin) in PEM polymerization buffer containing 1 mM GTP were added to ice-cooled cuvettes. These cuvettes were then placed in a water-jacketed cuvette holder in a diode array photometer (Beckman spectrophotometer DU7500) and rapidly warmed to 37°C. The absorbance at 350 nm in the presence and absence of drugs is proportional to the degree of tubulin polymerization.

### Transmission electron microscopy

MAP-enriched tubulin from porcine brain tissue (70% tubulin, 30% microtubule-associated proteins; Cytoskeleton, Inc.) was adjusted to 12 µM tubulin with PEM buffer containing 1 mM GTP and polymerized for 30 min at 37°C in the presence or absence of drugs. For negative staining, thin carbon support films were prepared by indirect sublimation of carbon on freshly cleaved mica. Samples were then absorbed to the carbon film and negatively stained with 2% (wt/vol) aqueous uranyl acetate (pH 4.5). After air drying, samples were examined in a Zeiss TEM 910 at an acceleration voltage of 80 kV and at calibrated magnifications using a line grid replica. Images were recorded digitally with a Slow-Scan CCD-Camera (ProScan, 1024×1024, Scheuring, Germany) with ITEM-Software (Olympus Soft Imaging Solutions, Münster, Germany).

## Supporting Information

Figure S1 **Chemical structures of tested tubulysin analogs and their GI_50_ values on L-929 cells.** GI_50_ values are given in bold and were determined in a tetrazolium salt (MTT) assay. L-929 cells were treated for 5 d and values present the average of two independent measurements. The numbering of tested analogs refers to Figure 1.
(TIF)

Figure S2 **HC analysis of γH2A.X fluorescence within the nuclear segment of U-2 OS cells.** The cells were treated for 48 h with antimitotic drugs at varying concentrations. For imaging, cells were fixed, γH2A.X was probed by immunofluorescence, and nuclei were stained with Hoechst33342. Nuclear segments were defined in Hoechst channel and intensity of phosphorylated H2A.X was calculated within these segments. Increasing intensities upon treatment with antimitotics is exclusively related to fragmented nuclei. The image in the upper left corner gives a detail of cells treated with the positive control 10 µM HU (hydroxyurea) and shows induced DSBs (DNA double-strand breaks) as determined by γH2A.X. Images were acquired on a BD Pathway 855 automated microscope and subsequently processed and analyzed in AttoVision v1.6.2. Bars represent the mean ± SEM of all cellular segments within a well.
(TIF)

Table S1 **Calculated percentage of HepG2 cells in G_1_, S, and G_2_/M phase of the cell cycle as determined by flow cytometry.**
(DOC)
