# Supplementary figures and images for: Pretubulysin: From Hypothetical Biosynthetic Intermediate to Potential Lead in Tumor Therapy
Source: PLoS One. 2012 May 17;7(5):e37416. doi: 10.1371/journal.pone.0037416 (PMC3355125; doi:10.1371/journal.pone.0037416)

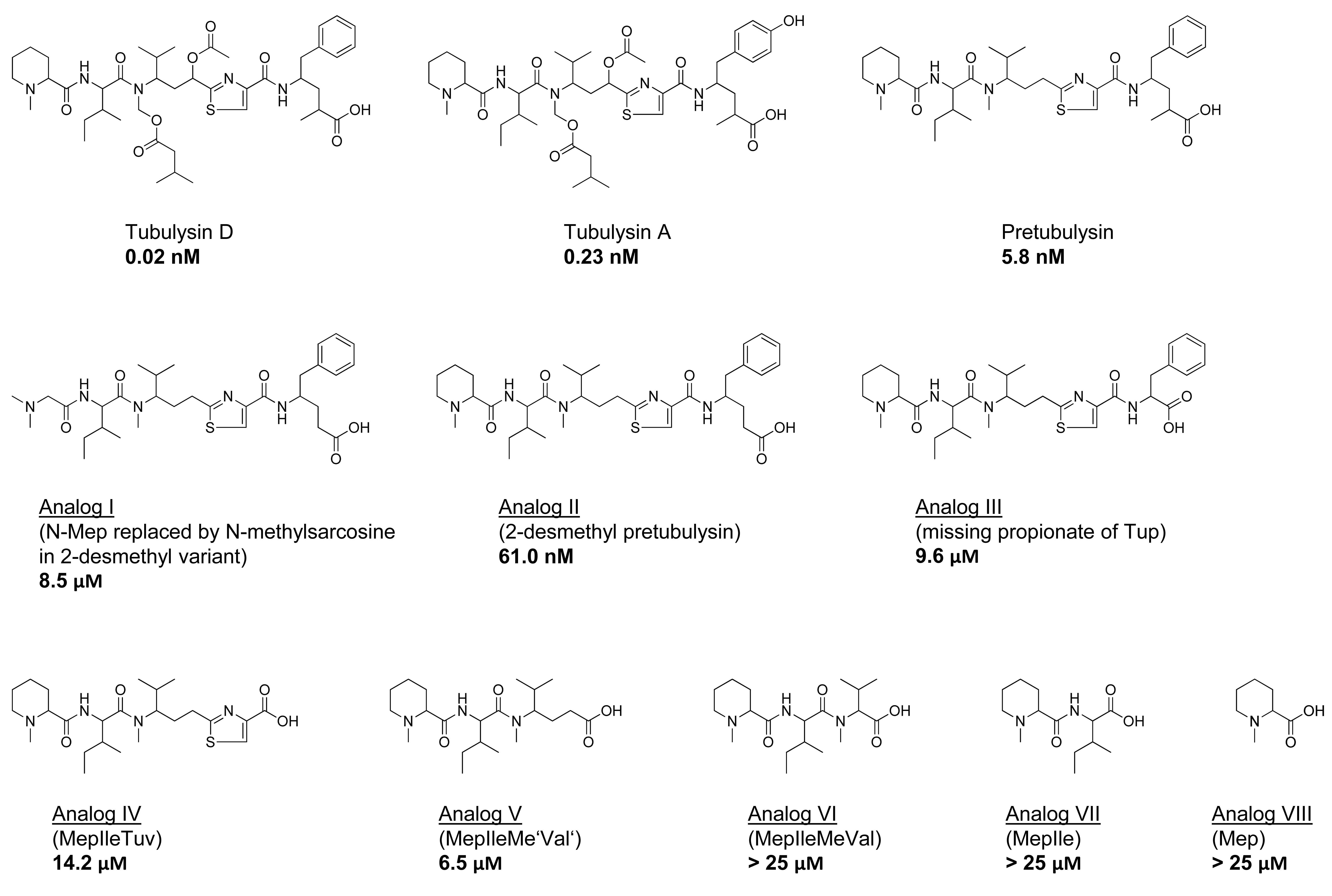

Supplement: Figure S1 — Chemical structures of tested tubulysin analogs and their GI50 values on L-929 cells. GI50 values are given in bold and were determined in a tetrazolium salt (MTT) assay. L-929 cells were treated for 5 d and values present the average of two independent measurements. The numbering of tested analogs refers to Figure 1. (TIF) [file pone.0037416.s001.tif]

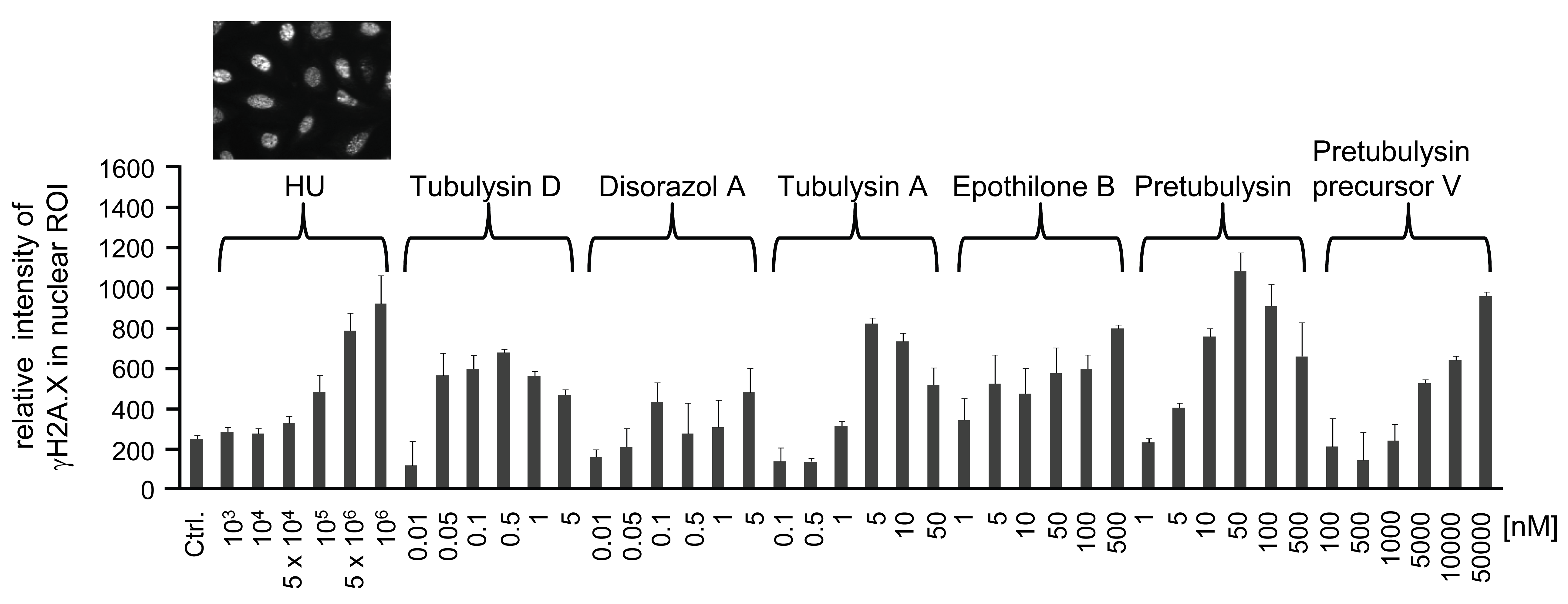

Supplement: Figure S2 — HC analysis of γH2A.X fluorescence within the nuclear segment of U-2 OS cells. The cells were treated for 48 h with antimitotic drugs at varying concentrations. For imaging, cells were fixed, γH2A.X was probed by immunofluorescence, and nuclei were stained with Hoechst33342. Nuclear segments were defined in Hoechst channel and intensity of phosphorylated H2A.X was calculated within these segments. Increasing intensities upon treatment with antimitotics is exclusively related to fragmented nuclei. The image in the upper left corner gives a detail of cells treated with the positive control 10 µM HU (hydroxyurea) and shows induced DSBs (DNA double-strand breaks) as determined by γH2A.X. Images were acquired on a BD Pathway 855 automated microscope and subsequently processed and analyzed in AttoVision v1.6.2. Bars represent the mean ± SEM of all cellular segments within a well. (TIF) [file pone.0037416.s002.tif]
