# Supplementary material for: Pretubulysin: From Hypothetical Biosynthetic Intermediate to Potential Lead in Tumor Therapy
Source: PLoS One. 2012 May 17;7(5):e37416. doi: 10.1371/journal.pone.0037416 (PMC3355125; doi:10.1371/journal.pone.0037416)
Supplement: Table S1 — Calculated percentage of HepG2 cells in G1, S, and G2/M phase of the cell cycle as determined by flow cytometry. (DOC) [file pone.0037416.s003.doc]

**Table S1. Calculated percentage of HepG2 cells^[a]^ in G_1_, S, and G_2_/M phase of the cell cycle as determined by flow cytometry.**

| **Sample^[b]^** | **G_1_** | **S** | **G_2_/M** |
| --- | --- | --- | --- |
| Control | 53.3 | 21.9 | 21.7 |
| 50 nM tubulysin A | 9.0 | 11.9 | 72.6 |
| 100 nM tubulysin A | 4.8 | 10.0 | 77.7 |
| 200 nM tubulysin A | 3.5 | 8.8 | 81.6 |
| 50 nM pretubulysin | 41.8 | 12.5 | 44.0 |
| 100 nM pretubulysin | 15.4 | 14.5 | 68.4 |
| 200 nM pretubulysin | 16.2 | 10.9 | 70.2 |
| 0.5 µM precursor V | 47.7 | 23.9 | 26.4 |
| 5 µM precursor V | 49.2 | 6.8 | 37.4 |
| 10 µM precursor V | 35.6 | 13.8 | 41.1 |
| 20 µM precursor V | 23.5 | 8.6 | 58.9 |

[a] Fixed cells were analyzed after PI staining on a Guava EasyCyte Plus cytometer and percentages were calculated by a Dean-Jett-Fox algorithm in FlowJo v7.6.5 software. [b] HepG2 cells were treated for 24 h with the assigned compound concentrations.
